# Manufacturing Signatures of Injection Molding and Injection Compression Molding for Micro-Structured Polymer Fresnel Lens Production

**DOI:** 10.3390/mi9120653

**Published:** 2018-12-10

**Authors:** Dario Loaldi, Danilo Quagliotti, Matteo Calaon, Paolo Parenti, Massimiliano Annoni, Guido Tosello

**Affiliations:** 1 Department of Mechanical Engineering, Technical University of Denmark (DTU), 2800 Kgs. Lyngby, Denmark; danqua@mek.dtu.dk (D.Q.); mcal@mek.dtu.dk (M.C.); guto@mek.dtu.dk (G.T.); 2 Politecnico di Milano, Department of Mechanical Engineering, 20156 Milan, Italy; paolo.parenti@polimi.it (P.P.); massimiliano.annoni@polimi.it (M.A.)

**Keywords:** manufacturing signature, process fingerprint, Fresnel lenses, injection compression molding, injection molding, micro structures replication, confocal microscopy, optical quality control, uncertainty budget, optimization

## Abstract

Injection compression molding (ICM) provides enhanced optical performances of molded polymer optics in terms of birefringence and transmission of light compared to Injection molding (IM). Nevertheless, ICM requires case-dedicated process optimization to ensure that the required high accuracy geometrical replication is achieved, particularly especially in the case of surface micro-features. In this study, two factorial designs of experiments (DOE) were carried out to investigate the replication capability of IM and ICM on a micro structured Fresnel lens. A laser scanning confocal microscope was employed for the quality control of the optical components. Thus, a detailed uncertainty budget was established for the dimensional measurements of the replicated Fresnel lenses, considering specifically peak-to-valley (PV) step height and the pitch of the grooves. Additional monitoring of injection pressure allowed for the definition of a manufacturing signature, namely, the process fingerprint for the evaluation of the replication fidelity under different process conditions. Moreover, considerations on the warpage of parts were related to a manufacturing signature of the molding processes. At last, the global part mass average and standard deviation were measured to correlate local geometrical replication performances with global part quality trends.

## 1. Introduction

Fresnel lenses are well-known optical devices with enhanced illumination properties combined with a compact and lightweight design. They are plano-convex optics, where the lens profile curvature is collapsed into a series of discontinuous frusto-conical grooves of reduced thickness. For mobile communication and electronic devices, as well as automotive and medical applications, the dimensions of the grooves lie in the micrometer scale and define the Fresnel lens optical performances [1,2,3,4]. Replication technologies represent the state-of-the-art solution to enable mass-manufacturing of polymer optics. The most cost-effective replication processes implemented currently in the industry are molding-based solutions such as injection molding (IM) and injection compression molding (ICM). Even though IM and ICM are established processes, it appears that they are still not clearly understood in the literature with regard to which process conditions provide the optimal results in terms of micro-geometrical replication for complex polymer optical systems.

**Figure 1:**
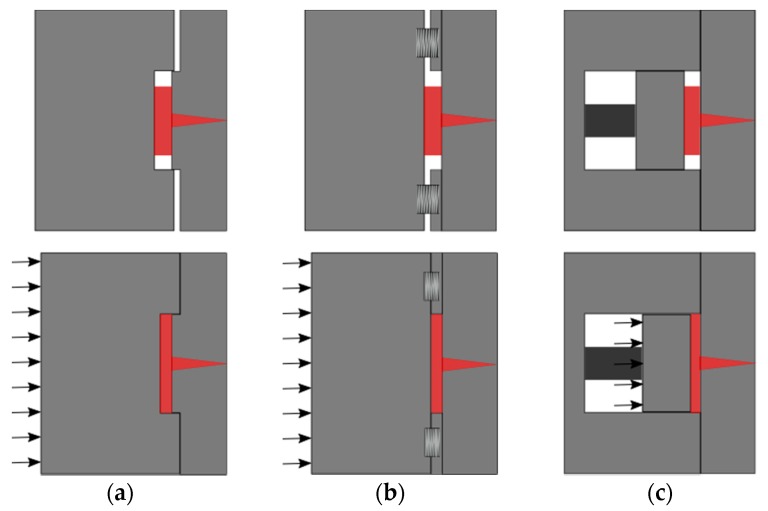
Different injection compression molding (ICM) mold closures before (upper picture) and during compression (lower picture), and their schematic architectures: (**a**) generating a “vertical flash” area; (**b**) using a spring-connected “compression-frame”; (**c**) adopting an actuated compression die.

Despite IM and ICM being interrelated processes, an additional phase in the operations sequence of ICM, i.e., compression, may have a substantial impact on the final product. In fact, while in IM, the polymer melt is injected in a closed mold cavity with almost the same dimensions and geometry of the final part, depending on the material shrinkage (i.e., the ones achieved when the two halves of the mold are forced against each other by the clamping force provided by the IM machine), in ICM, the melt is injected into an “open” cavity with the two mold halves initially being separated from each other. The mold is successively closed during a compression phase at the end of the operations sequence or during the injection phase [5]. The additional gap between the molds is called the compression gap, and it provides the necessary stroke to perform the compressing action. The compression gap is achieved in different ways. One of them consists of the design of molds with a so-called “vertical flash” area (see Figure 1a), in which the entire mold halves are kept separated. Another option employs a “compression frame” into the mold. The frame is built with spring systems, and an additional compression plate is mounted into the movable mold side (see Figure 1b). A more accurate but also more cost-intensive solution consists of the adoption of an independent “compression core” (see Figure 1c). In this configuration, the insert in the movable cavity is directly actuated for cavity closure, and it performs the compression. The selection of the most suitable solution depends on cost, and partly on geometry and target accuracy. The compression action is seen as an additional holding phase that is applied to the material inside the cavity. In IM, holding starts at the so-called switch/over point, i.e., the moment when part filling is considered complete, and the machine control switch from a filling control criterion (injection velocity, screw position, injection pressure, etc.) to a holding control criterion, generally the holding pressure. Holding ensures that the final part volume is equal to the one of the cavity, since the polymer material shrinks during cooling. In IM, holding is consequently a crucial quality step; however, for parts showing long flow length and/or small wall thickness, the required holding pressure to compensate for the pressure drop can be significantly high, as are the resulting residual stresses inside the part. The major advantage of ICM consists of the opportunity to reduce stresses in the part, as compression action provides an in-thickness holding effect on the cavity, ensuring a uniform distribution of stresses inside the cavity, while the part solidifies [6,7,8]. For this reason, ICM has been extensively favored to IM in the production of optical components. It is proven that it provides enhanced optical functionalities by reducing the birefringence and increasing the transmission efficiency, which are both correlated to the part internal stresses distribution. Birefringence effect is an optical property, which causes preferential light multiple refractions within the optics [9,10,11]. At the same time, the light transmission measures the effective spectral and power transmittance of the optics, i.e., a combined result of the material absorption of specific wavelengths, reflection, and surface loss. All of these aspects are heavily dependent on the geometrical and dimensional optical design, and on local surface defects such as roundings or drafts that are natural outcomes of the molding process [12,13]. Even though ICM leads to functional benefits, the compression phase increases IM complexity, because additional parameters must be taken into consideration for optimizing the process. Suzuki et al. [14] presented the importance of increasing the compression stroke in order to improve surface replication. On the contrary, Rohde et al. [15] discouraged the increment of the compression gap, as it reduces the transcription ratio of micro-structures. These controversial results in relation to the compression gap require further consideration, and they have received attention in recent research. Masato et al. [16] observed a significant interaction between the compression gap and the injection velocity, confirming that the optimization of the compression gap must take into account the overall polymer flow conditions. The study highlighted the negative impact of a large compression gap with respect to the replication homogeneity. A similar result was achieved by Chen et al. [17], who proved that a smaller compression gap induces a more uniform part shrinkage, and in another study, Chen et al. [18] validated that a larger compression gap increases part birefringence. The different theories regarding the compression gap selection can be justified by the results obtained by Shen et al. [19]. In their work, replication and birefringence improvements were initially noticed by increasing the compression gap. Such a gain was due to a larger compression energy provided to the polymer melt that increased the shear rate and reduced the viscosity. However, the advantage arose from a delay in the compression phase, due to either a large compression gap, or a slow compression speed (or both), that increased the material viscosity as the polymer cooled down before being compressed, generating heat dissipation with the mold and shear rate reduction. In these conditions, the polymer melt front formed a thick solid layer, so-called “skin layer” on the mold wall, limiting the melt flow more than what the compression could favor. This theory was supported by Ho et al. [20] observing an injection pressure and shear rate reduction with increased compression strokes. An additional result on compression parameters was given by Ito et al. [21], who found that the compression starting point and the compression gap are relevant factors affecting both optical performance and internal stresses. The optimal compression start was identified when the cavity injection was completed. The work of Kuo et al. [22] is one of the few studies mentioning the importance of the compression force. In the study, it was found that both IM and ICM parameters affect the replication of micro-features less when the compression force exceeds a certain threshold. Sortino et al. [23] verified the influence on the transcription ratio of IM factors such as holding pressure, injection velocity, and mold temperature in ICM. Their study demonstrated that the statistical effect of the IM factors was reduced in ICM. A confirmation was given by Han et al. [24], who discovered that the holding pressure could be reduced in ICM up to 50% with respect to IM, thanks to a more uniform cavity pressure distribution that is achieved with the compression phase. In the case of micro- or nano-structures replicated by ICM, a significant effect on the replication is also given by the mold temperature, as proved by Rytka et al., Nagato et al., and Chuan-Zhen et al. [25,26,27]. The effective local mold temperature also supports different replication quality conditions in dependence on the cavity design and the polymer melt flow [28,29,30]. Understanding the effective replication behavior of surface grooves is of paramount importance, to ensure the designed functionality of polymer optics, such as Fresnel lenses. Moreover, the complexity of the lenses’ features demands for dedicated quality control criteria. For example, the so-called “interference by adjacent step” is a Fresnel lens efficiency, loss due to its stepped discontinuous profile [31]. In some cases, it is possible to reduce the efficiency loss by designing total internal reflection (TIR) lenses [32,33,34]. However, molding-based processes are not always capable of reproducing the ideal design, e.g., because of minimum draft angles required for de-molding [1]. In addition, the sharp edges of the micro-stepped grooves cannot be fully replicated, producing rounded features that reduce the overall optical performance [35,36,37]. In general, functional optical tests based on photogrammetry ensure the correct optical functionality. Such tests are robust and investigate whether optical aberrations occur while operating the lens. From those results, it is possible to reconstruct the lens geometry when an optical model is available. Nevertheless, such tests do not distinguish whether aberration occurs due to material dependent degradation or geometrical/dimensional imperfections occurring in the manufacturing process. The identification of manufacturing signatures, i.e., the link between a measurable feature of the final part geometry and the individual manufacturing process conditions, allows for a comprehensive understanding of the production steps, and ensures effective and efficient optimization solutions [38,39].

Dedicated geometrical metrology is needed for the assessment of a manufacturing signature. Tactile measuring equipment is still extensively exploited, even though they can generate scratches on the lenses surface and generally require long set-up time and suffer accuracy loss in PV measurements [40,41]. Alternatively, non-contact optical solutions such as 3D optical microscopes can also be adopted for the scope [42]. Nevertheless, the high transparency of the material prevents the possibility of using focus variation systems or contrast-based microscopes. Similar limitations of these techniques are observed when optical or near-optical surface roughness (i.e., down to single digits to tens of nanometers, respectively) is measured [43,44]. In addition, setting up a scatterometry-based inspection is challenging, as the tips and roots of the Fresnel surface severely manipulate the scattering properties of the specimen [45]. In this study, the low aspect ratio surface micro-grooves of a Fresnel lens were investigated using a confocal microscope. The microscope principle is well known for its flexibility and the possibility to have both lateral and vertical resolutions in the sub-micrometer level. In this work, the identification of different manufacturing signatures in the production of Fresnel lenses is tackled. To do so, an initial metrological procedure with a detailed uncertainty budget is proposed, to evaluate the lens surface micro-feature replication. The methodology is proposed for two different materials, providing robust applicability for the procedure. The objective of this work is to provide a comprehensive methodology for the quantitative evaluation of IM and ICM performances, based on manufacturing signatures that address micro-replication quality. The four different manufacturing signatures (micro-replication accuracy, warpage, injection pressures, and part mass) were applied, providing a methodology for the optimization of IM and ICM. These four manufacturing signatures are employed, and their respective results are compared simultaneously as drivers of the optimization process for micro-structured optical parts manufacturing. The methodology, based on a metrological approach, provides a robust guideline for the effective molding of high precision polymer optics. 

## 2. Materials and Methods

### 2.1. Device under Investigation

**Figure 2:**
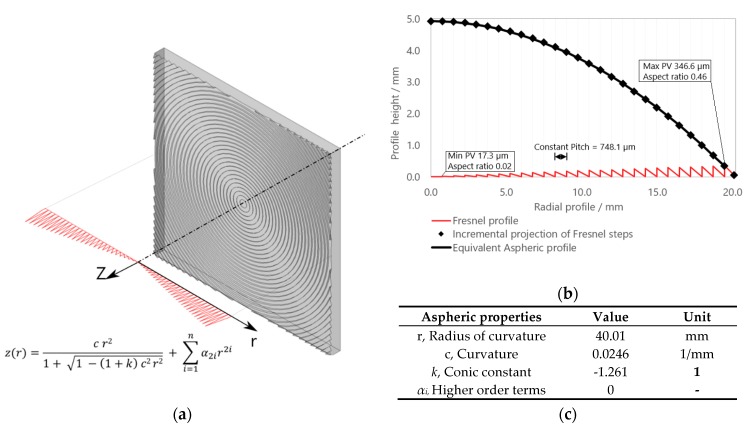
Representation of the studied Fresnel lens: (**a**) 3D view of the flat, squared, 40 mm × 40 mm Fresnel surface aperture, with a section view of the axis-symmetric radial profile, which follows a stepped aspheric profile as described by the conic equation; (**b**) a highlight of the section profile in the *z*, *r* plane with indication of the peak-to-valley (PV) nominal specifications in comparison with the equivalent continuous aspheric curvature; (**c**) summary table of the aspheric properties of the considered lens.

**Figure 3:**
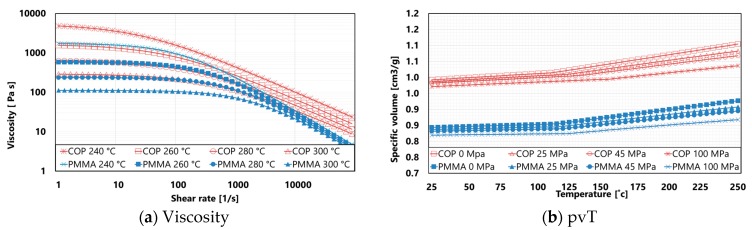
Cyclo-olefin polymer (COP) and polymethyl methacrylate (PMMA) material properties: (**a**) shear rate-dependent viscosity at different temperature values; (**b**) temperature-dependent specific volumes at different pressure values. Data collected from the Moldflow^®^ software database, version 2018, by Autodesk^®^ (San Rafael, CA, USA).

The component under investigation is the aspheric-corrected square Fresnel lens shown in Figure 2. Tolerance specifications are allocated to surface groove peak-to-valley (PV), as presented in a previous study [46]. The materials employed for the experimentation were a cyclo-olefin polymer (COP) commercially available under the trade name Zeonex^®^ E48R, produced by Zeon© company, Tokyo, Japan, and a polymethyl methacrylate (PMMA), traded under Altuglas^®^ V825T, produced by Arkema©, Colombes, France. Their viscosities (a) and pressure-specific volume-Temperature (pvT) curves (b) are reported in Figure 3. The data were collected from the Moldflow^®^ software database, version 2018, by Autodesk^®^ (San Rafael, CA, United States).

### 2.2. Injection Molding and Injection Compression Molding Machine 

**Figure 4:**
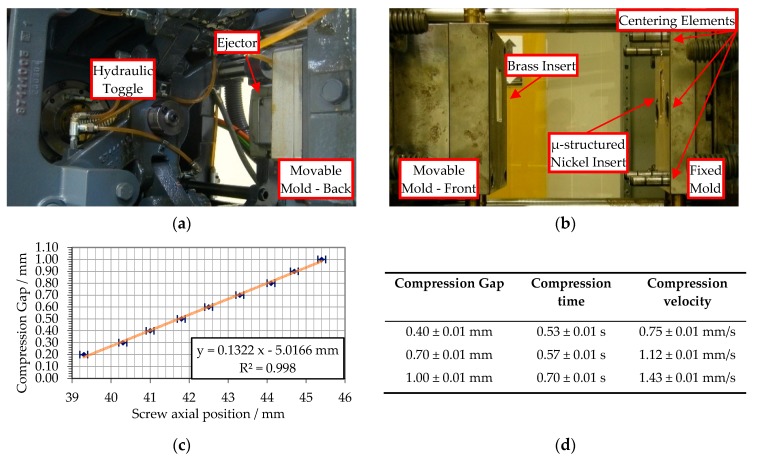
Injection compression molding solution: (**a**) toggle clamp unit with its hydraulic circuit; (**b**) movable (left) and fixed (right) mold plates in open configuration; (**c**) linear regression of the toggle unit position against the mold plates’ closure to control the compression gap; (**d**) summary table of the compression gap, compression time, and average compression velocity.

Experiments were performed on an injection molding machine (Negri Bossi©, Cologno Monzese, Milano, Italy) equipped with a reciprocating injection screw having a diameter of 32 mm, capable of a maximum clamping force of 600 kN. The clamping unit was equipped with the toggle clamp system represented in Figure 4a. The unit had a control on the position of the toggle with a repositioning error of 0.1 mm. The closure between the fixed and movable mold plates was measured at the two different positions of the toggle. In Figure 4c, a strong linear correlation (R^2^ = 99.8%) was found between the toggle positions and the mold halves measured the closure with a resulting precision on the gap between the plates corresponding to 0.01 mm. In this way, the compression gap was controlled with the same level of precision, which is of central importance since the closing action of the mold compresses the melt inside the cavity. Furthermore, the compression speed is limited by the machine dynamics, and different average compression velocities and times for different starting compression gaps are reported in Figure 4d.

### 2.3. Metrology and Uncertainty

**Figure 5:**
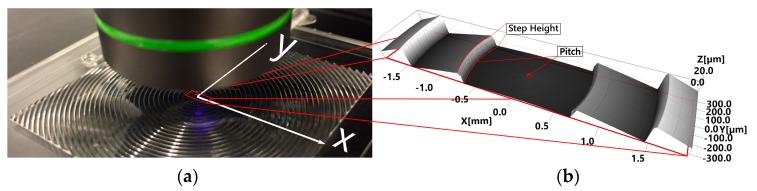
3D measurements performed with the laser scanning confocal microscope: (**a**) Measurement set-up using the 20× objective; (**b**) Resulting 3D view of the sampled image.

The employed confocal instrument was a commercially available microscope with the trade name Lext OLS4000, manufactured by Olympus, Tokyo, Japan. Measurements were performed in the central location of the lens, as shown in Figure 5, using the microscope 20× standard magnification objective (measurement parameters summarized in Table 1). The lens showed a global squared dimension of 40 mm × 40 mm. The center of the lens was selected, as its near contour allowed for the evaluation of warpage and replication differences, depending on the features geometry, symmetry, and location with respect to the lens center (where the optical axis lies). Stitching of five different images was executed with a 3.712 mm × 0.640 mm sample area of the lens center. In this area, the first groove of the Fresnel lens was assessed. The groove had the lowest aspect ratio in the lens (17.3 µm/748.1 µm); nonetheless, the feature is the shortest in the lens design. The stitching overlapping factor between each image was 20% of the area of a single scan. An image size of 5328 × 913 pixels was achieved, with a resolution below the diffraction limit for the X-axis and above it for the Y one. The overall expanded uncertainty related to measurements was guided by ISO 15530-3:2011 [47] and ISO 14253-2:2007 [48]. The individual uncertainty contributors are reported in Table 2 for step height, and in Table 3 for pitch measurements, and they were calculated as described in a previous study [46]. The traceability was established through calibrated gauge blocks. A step height of (14.45 ± 0.26 µm) was obtained wringing together two gauge blocks, and it was related to the height of the lenses’ grooves, while a gauge block with a value of 1500 ± 0.08 µm was related to the pitch measurements. In this last case, the measurements were made by stitching three fields of view in the X direction, both for the measured lenses and the reference standards.

### 2.4. Design of Experiments 

The injected polymer volume and dosage were calibrated with preliminary injection molding short shots experiments, considering a screw injection velocity of 40 mm/s. An optimal switchover was defined for an injection screw position of 10 mm from the end stroke position. The switchover point was kept constant for both COP and PMMA in all process conditions. For all the experiments, the compression phase started after the injection, i.e., when the reciprocating screw reached the optimal switchover position of 10 mm. An initial factorial experimental campaign was performed to investigate the interaction between compression and holding phase, with the aim of analyzing which of the two process phases had a larger influence on the manufacturing signature, for the two analyzed materials:

a. IM without holding pressure;
b. IM with holding pressure;
c. ICM without holding pressure;
d. ICM with holding pressure.

Compression and post-filling holding phases were alternately switched on and off. Table 4a presents the factorial design. In this case, the compression gap was kept constant at 0.7 mm. All of the other process parameters are presented in Table 4b. A further optimization design was performed for the case of COP material based on the initial experimental campaign results. Aiming to validate the possibility to use the methodology as optimization tool of process parameters settings in ICM, only COP was chosen, as the two materials had different viscosity and processing requirements. Compression gaps and holding pressure levels were varied from 0.4 mm to 1.0 mm, and from 250 bar to 450 bar respectively, as shown in Table 5a. The compression starting point, switchover, and injection velocity were not changed, and kept as in the previous experimentation (see Table 5b). Thermal conditions such as melt and mold temperatures were not varied through the two experimental sessions. The melt temperature for the COP and PMMA materials was set respectively at 280 °C and 260 °C, being the viscosity of the material that was comparable at low shear rates for those temperature values.

The mold’s temperature controller was set according to previous experimentation and the material manufacturer suggested values. The measured mold temperature was constant on both fixed and movable sides during the whole experimentations, being (105 ± 3) °C for COP and (93 ± 3) °C for PMMA. The temperature of the mold was not changed, and considered an experimental constant parameter, as it strongly affects the process cycle time.

## 3. Results

Ten different runs were performed, and three samples were extracted, measured, and averaged for each condition. The quality control was performed on the absolute dimension measurements of the pitch and peak-to-valley (PV) step height of the Fresnel lens’ central grooves. The warpage was investigated, considering the central profile of the sampled images. Injection pressure results were analyzed separately for the filling and holding phases. Average and mass standard deviations are presented as part of global quality features.

### 3.1. Absolute Dimensions

**Figure 6:**
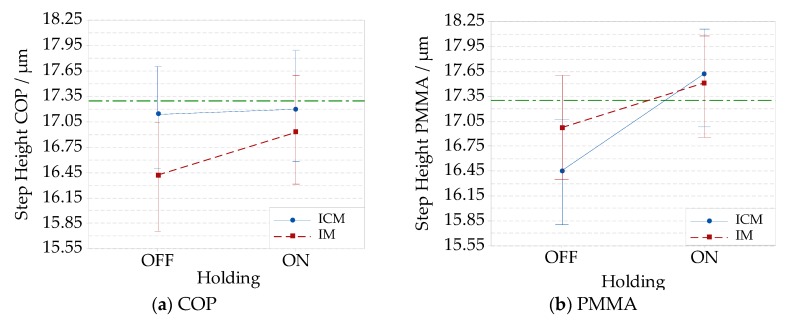
Interaction plots showing the replication of absolute dimensions of the Fresnel lens’ grooves with a nominal step height of 17.3 µm. A measurement uncertainty is added to the results obtained for IM without the holding pressure, IM with holding pressure, ICM without holding pressure, and ICM with holding pressure: (**a**) COP material; (**b**) PMMA material.

Average absolute dimension measurements and uncertainties are reported in the form of interaction plots. When analyzing the step height (Figure 6a), it is possible to observe that for the case of IM without holding, the use of compression provided a higher step height replication for the COP material. On the contrary, the holding phase increased the step height replication in ICM for PMMA, as shown in Figure 6b. The other shown conditions are statistically equivalent, due to the relatively high measurement uncertainty with respect to the process deviations. In the case of the COP step height, the absolute deviations from nominal specifications ranged from a maximum value of (1.5 ± 0.7) µm, in the case of IM without holding, to a minimum of (0.1 ± 0.7) µm in the case of ICM.

**Figure 7:**
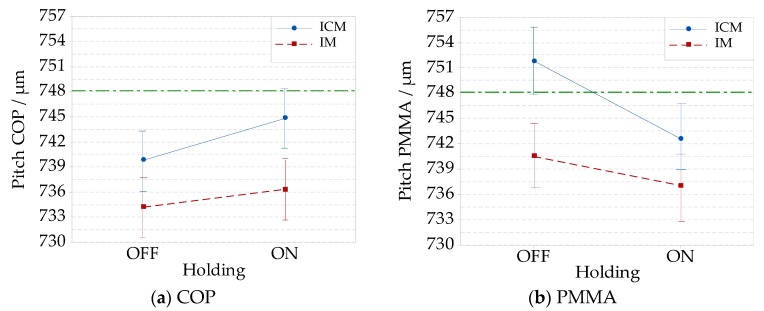
Interaction plots showing the replication of the absolute dimensions of Fresnel lens’ grooves with a nominal width of 748.1 µm. The measurement uncertainty is added to the results obtained for IM without holding pressure, IM with holding pressure, ICM without holding pressure, and ICM with holding pressure: (**a**) COP material; (**b**) PMMA material.

**Figure 8:**
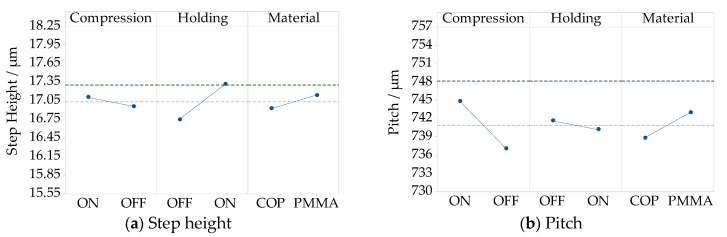
Main effects plot of compression, holding, and material on the average replication of the absolute dimensions of a Fresnel lens’ groove with a nominal height of 17.3 µm and width 748.1 µm: (**a**) effects on step height; (**b**) effects on pitch.

For PMMA, absolute deviations from nominal specifications are at a maximum value of (0.8 ± 0.6) µm in the case of ICM without holding, and at a minimum of (0.1 ± 0.6) µm in the case of IM. Considering the measured values, the compression improved the pitch replication for both COP and PMMA material, as shown in Figure 7a,b. The holding effect was significant and increased the pitch average only for COP in ICM. When employing PMMA, an opposite effect of holding was observed, as shown in Figure 7b. It reduced the average pitch in ICM with statistical significance. In this case, the reduction of pitch was attributed to the higher part of the shrinkage that the PMMA samples underwent in comparison to COP for the considered processing conditions. For all these conditions, the COP maximum absolute deviations from the target values were (13.9 ± 3.8) µm in case of IM without holding, which reduced to a minimum value of (3.3 ± 4.4) µm for ICM. Analyzing PMMA, the absolute maximum deviation from the nominal value was (11.1 ± 4.2) µm for IM, and the minimum one (3.7 ± 4.2) µm for IM without holding. The experimental factors’ effects are plotted into the main effect plots in Figure 8a for step height, and Figure 8b for pitch. The addition of compression to IM led to improvements of both pitch and step height replications. PMMA showed an average higher replication than COP, which can be justified by its lower viscosity at the same processing temperature and pressure. The holding phase was responsible for a higher step height replication of the features, while the pitch of the grooves was more dependent on the material and compression.

**Figure 9:**
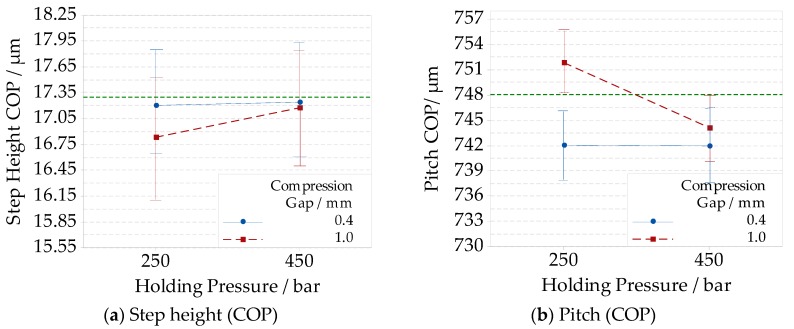
Interaction plots showing the replication of absolute dimensions of the Fresnel lens’ groove with a nominal height of 17.30 µm and a width of 748.10 µm. The measurement uncertainty is added for the optimized ICM on compression gaps and holding pressures levels for the COP material: (**a**) to the step height; (**b**) to the pitch.

A further analysis regarding the interaction between the holding pressure and the compression gap was carried out in the optimization experimental campaign for the COP material. The related results are reported in Figure 9a for step height and Figure 9b for pitch. At a high holding pressure (450 bar), the replication of the step height and pitch was not affected by the compression gap level.

As shown in Figure 9a, the absolute deviation from the nominal step height is equal to (0.1 ± 0.7) µm for both compression gap levels. For the replicated pitch (see Figure 9b), the absolute deviation was (6.2 ± 4.4) µm with a 0.4 mm compression gap, and (4.1 ± 4.4) µm with a 1.0 mm compression gap. When the holding pressure was lower (250 bar), the effect of the compression gap was higher for both pitch and step height replication. The results also show another trend, that when compression gap was kept at a low level (0.4 mm), the replication difference related to the holding pressure levels was negligible. The reduction of pitch was attributed to the lower shrinkage occurring at high holding pressure level. These combined results indicated that the compression gap and the holding pressure levels should not be considered as independent factors, and that they should be optimized simultaneously to ensure higher replication quality.

### 3.2. Warpage

Form deviations, named as warpage, are generally associated with uneven cooling and differential shrinkage of the part, as well as deformations induced by the demolding of the part from the mold. In the present case, warpage attributed to unbalanced shrinkage was caused by different process settings, and was investigated along the orthogonal direction with respect to the main symmetry axis of the part, i.e., orthogonally to the melt flow direction (left/right).

**Figure A1:**
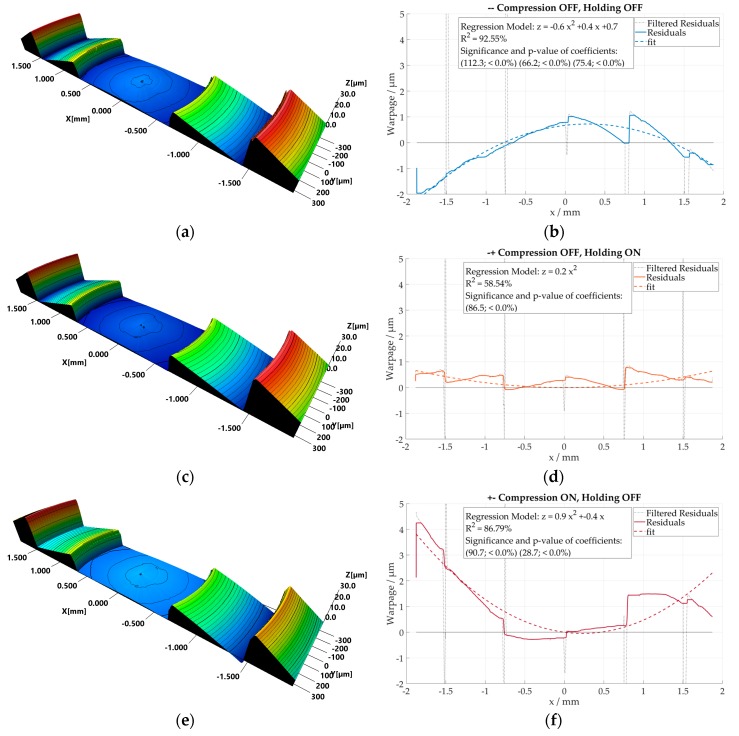

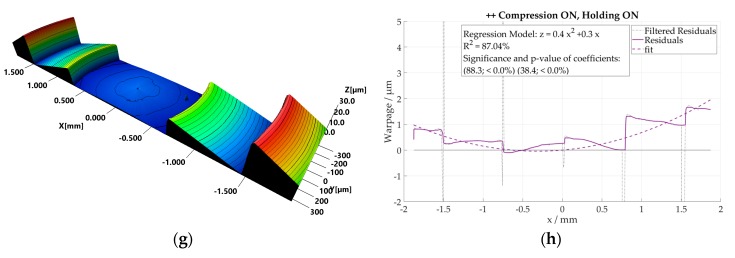
On the left, 3D view of the lens’ central location, sampled as shown in Figure 5. On the right, the residuals of the respective x profiles in comparison with nominal geometry, sampled in the image center. The following process conditions are considered for the COP material results: (**a**,**b**) IM without holding; (**c**,**d**) IM; (**e**,**f**) ICM without holding; (**g**,**h**) ICM.

**Figure A2:**
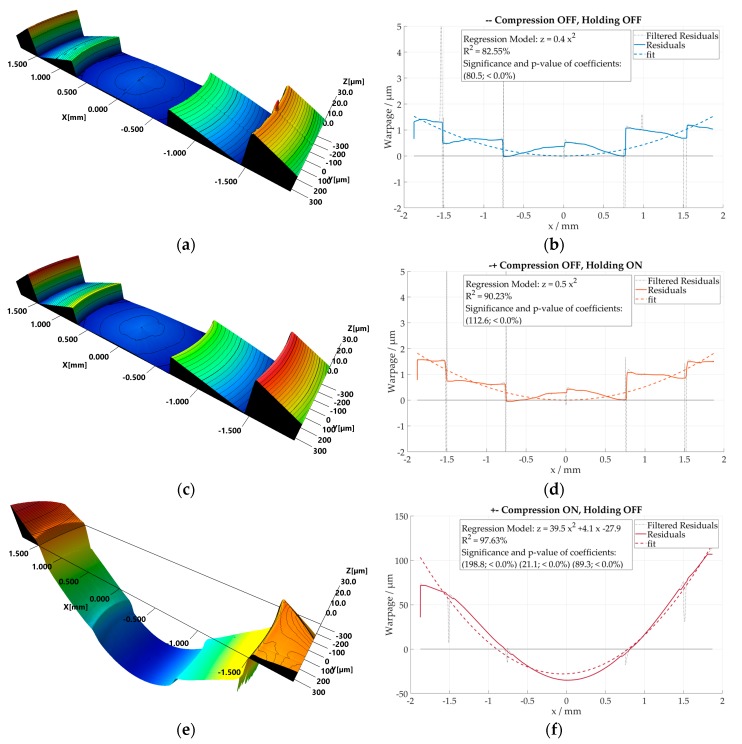

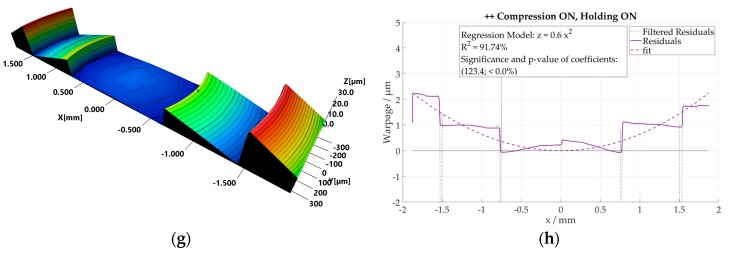
On the left, 3D view of the lens’ central location, sampled as in Figure 5. On the right, the residuals of the respective x profiles, in comparison with nominal geometry sampled in the image center. The following process conditions are considered for PMMA material results: (**a**,**b**) IM without holding; (**c**,**d**) IM; (**e**,**f**) ICM without holding; (**g**,**h**) ICM.

**Figure A3:**
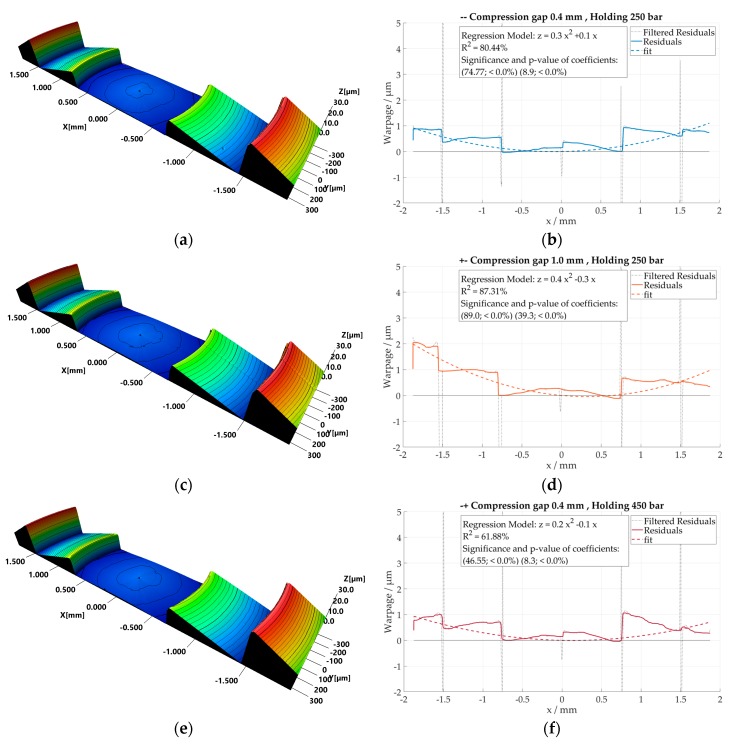

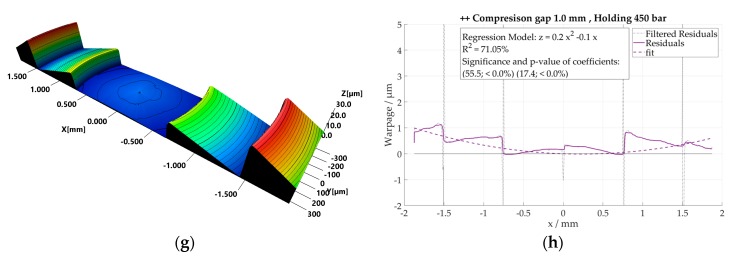
On the left, 3D view of the lens’ central location sampled as in Figure 5. On the right, the residuals of the respective x profiles in comparison with nominal geometry, sampled in the image center. The following process conditions are considered for COP material results: (**a**,**b**) Compression gap 0.4 mm and holding pressure 250 bar; (**c**,**d**) Compression gap 1.0 mm and holding pressure 250 bar (**e**,**f**) Compression gap 0.4 mm and holding pressure 450 bar; (**g**,**h**) Compression gap 1.0 mm and holding pressure 450 bar.

Warpage as a manufacturing signature on the produced Fresnel lenses was analyzed on the residuals from the nominal profile geometries (Figure A1, Figure A2 and Figure A3 in Appendix A). Residuals were filtered using a median filter, and fitted with second-order polynomials. The selection of the degree of the polynomials was made on the joint inspection of the coefficients of determination (R^2^) and the significant coefficients in the regressions of several-order polynomials, eventually choosing the best behavior. Warpage individual results are reported in Table 6.

**Figure 10:**
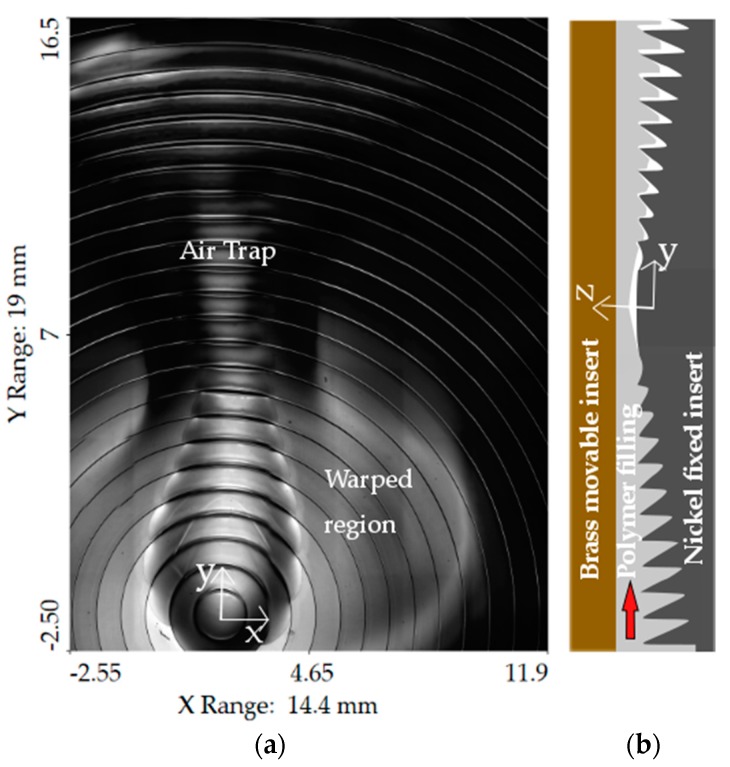
Combination of warpage and air trap in the replication of PMMA in ICM without holding: (**a**) Top view of the defect in the central location of the lens; (**b**) schematic visualization of air trap formation inside the mold cavity during the filling phase.

In the first analysis, for the COP material, a maximum absolute deviation of 4.3 µm was observed for the case of ICM without holding (Figure A1f); for the case of PMMA, the maximum observed deviation was 106.9 µm again for the case of ICM without holding (Figure A2f). In this last case, the form error was not the only defect associated with the part. A further analysis showed that the combined effect of warpage and of an air trap occurred in the central region of the lens. ICM without holding produced the lower form of replication and favored air traps during the part filling. In this case, a higher initial cavity volume ensured the possibility of performing compression; nonetheless, this fact resulted in a higher cavity pressure drop to produce the same filling conditions (as shown in Section 3.3. The shape of the Fresnel lens’ grooves promoted the air trap, starting from the lens’ center, where the slopes of the grooves changed with respect to the melt front direction, and the air stagnation area was favored (Figure 10).

Warpage was also inspected by observing the quadratic regression coefficients. Since they reduced when considering IM instead of ICM, in the first experiments, compression favored a less stable shrinkage of the parts. This behavior can be recognized in Figure A1d,h for COP, and Figure A2d,h for PMMA, respectively.

When ICM was optimized using a high holding pressure level (450 bar), for both compression gap levels (0.4 mm–1.0 mm), the quadratic regression coefficients were at a minimum value of 0.2 µm/mm^2^ (Figure A3f,h), indicating that warpage could be minimized in ICM with respect to IM. In the optimization case, the effect of compression gap was recognizable only for a low holding pressure (250 bar), as the coefficient increased from 0.3 µm/mm^2^ in the case of a low compression gap (0.4 mm) (Figure A3b), and to 0.4 µm/mm^2^ in the case of a long compression gap (1.0 mm) (Figure A3d).

The interaction of the two factors supports the previous observations, and is in accordance with the step height results described in Section 3.1 by (Figure 9a). Finally, the case of a larger compression gap resulted in a greater pressure drop in the cavity (Section 3.3), not only reducing the absolute replication of the features, but also increasing the warpage of the parts.

### 3.3. Injection Pressure

**Figure 11:**
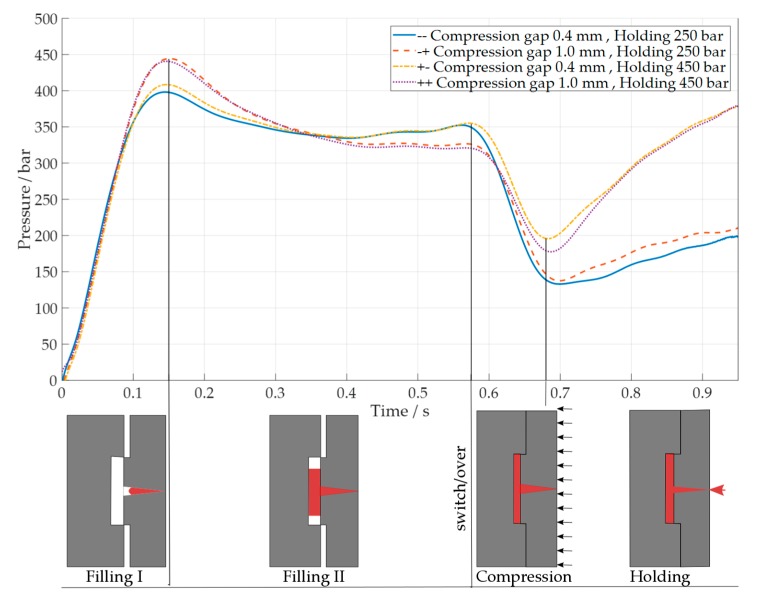
Injection pressure over time for a two-level compression gap (0.4–1.0 mm) and holding pressure (250 bar–450 bar). Compression gap of 1.0 mm and holding pressure of 250 bar; compression gap of 0.4 mm and holding pressure of 450 bar; compression gap of 1.0 mm and holding pressure of 450 bar: before and after switch/over.

Injection pressure profiles over time were investigated as fingerprints of the different process settings [49]. Plastic pressure was measured in the injection chamber before the nozzle with a pressure transducer (Dynisco^®^ Europe GmbH, Heilbronn, Germany, model MDT465C) and a sampling rate of 1 kHz. This pressure was equivalent to the pressure of the hydraulic circuit that moved the ram and was displayed in the machine control user display, and it is shown in Figure 11. The pressure was integrated over time (*P_work_*) on a defined time period, according to Equation (1):

*P_work_* = ∑*_i_*_=1:*n*−1_ · (P*_i_* + P*_i+_*_1_) · Δt · 1/2 (1)

where *n* is the total number of sampled pressure points, P*_i_* and P*_i+_*_1_ are consecutive injection pressure values, and Δt is the pressure sampling time.

**Figure 12:**
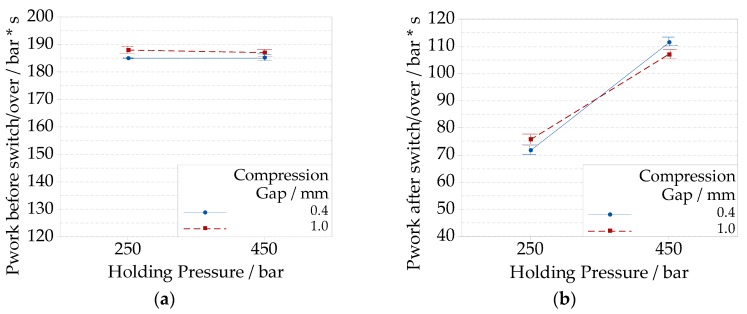
Interaction plots of the integral of pressure over time for the different compression gap and holding pressure levels: (**a**) calculated on a time interval that goes from zero to end of filling (before switch/over); (**b**) calculated on a time interval that goes from the end of injection until end of compression (after switch/over).

*P_work_* was calculated in two parts, one before (Figure 12a) and one after (Figure 12b) the switch/over point, which corresponded to an injection time of 0.57 s. Only an initial part of the holding phase was considered, up to an injection time of 0.95 s. The results are presented only for the optimization experiments in ICM using the COP material. Different compression gap levels modified the shapes of the pressure curves before the switchover. During the initial part of the filling, the pressure reached the maximum value depending on the compression gap level. A longer compression gap (1.0 mm) induced a greater pressure drop, considering the constant injection speed. The maximum injection pressure rose from (398.2 ± 3.7) bar and (408.5 ± 3.8) bar for the low compression gap (0.4 mm), to (444.4 ± 3.4) bar and (440.7 ± 3.5) bar for the high compression gap (1.0 mm), respectively, for low (250 bar) and high holding pressures (450 bar). After this point, the pressure started to decrease as the cavity was further filled. In this case, a higher compression gap also sustained a greater pressure drop. The pressure reached a value at the switchover point of (351.4 ± 3.7) bar and (355.2 ± 3.8) bar for the low compression gap (0.4 mm), which further reduced to (326.7 ± 3.4) bar and (321.0 ± 3.5) bar for a high compression gap in conditions of low and high holding pressures, respectively.

The integral of the curves over time (*P_work_*) decreased for the low compression gap from (188.0 ± 0.1) bar·s and (187.1 ± 1.3) bar·s to (185.5 ± 1.4) bar·s and (185.2 ± 1.3) bar·s for low and high holding pressure respectively. The overall integration of pressure over time demonstrated that the major contributor of the pressure during filling was given by the compression gap before the switch/over point as cavity size was changed. The interaction plot in Figure 12a shows this result.

After the switch/over, *P_work_* decreased from (107.1 ± 2.2) bar·s and (111.4 ± 2.2) bar·s in the case of high holding pressure to (75.7 ± 1.7) bar·s, and (71.7 ± 1.5) bar·s for the low holding pressure level, respectively, for a high and low compression gap. The transient from velocity to pressure control can be seen in Figure 11, as the molding machine increases the pressure to the desired holding control level with a certain lag.

In Figure 12b, the interaction between holding pressure and compression gap levels showed that the compression had a contribution, which depended on the pressure condition at the switchover, and on the energy that is introduced by the compression itself. Nonetheless, this contribution was 10 times lower than the one was given by the holding pressure control, as shown in the last part of Figure 11. As *P_work_* could be considered as a direct indicator of the energy stored in the polymer during processing [49]. The maximum energy was achieved when the holding pressure was set at a high level (450 bar) and the compression gap was set to a low level of 0.4 mm.

### 3.4. Part Mass

**Figure 13:**
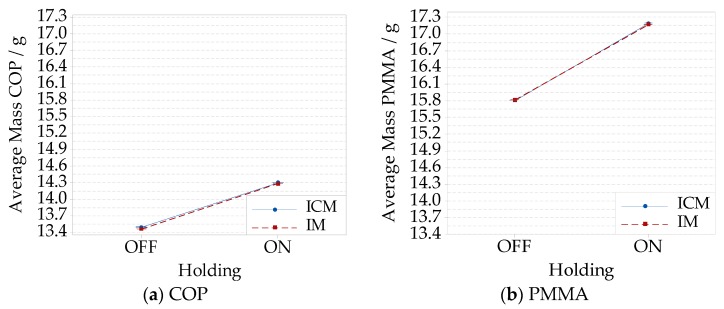
Interaction plots showing the average part mass obtained for IM without the holding pressure, IM and ICM without the holding pressure, and ICM for: (**a**) COP material; (**b**) PMMA material.

The parts’ mass was measured on all the 10 specimens per process condition, including the sprue. The interaction plots of the average part mass highlighted that a major effect on the average part mass was given by the holding phase for both COP (Figure 13a) and PMMA (Figure 13b). For COP, in case of IM, the average part mass increased from (13.463 ± 0.024) g to (14.283 ± 0.003) g when holding was performed, and from (13.493 ± 0.014) g to (14.298 ± 0.011) g in the same case for ICM. Considering PMMA, the increment was from (15.808 ± 0.033) g to (17.168 ± 0.021) g in IM, and from (15.803 ± 0.036) g to (17.187 ± 0.050) g for ICM.

**Figure 14:**
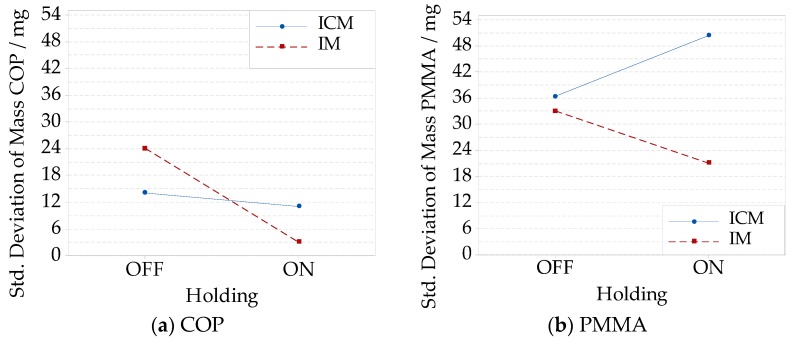
Interactions plots showing the part mass’ standard deviation for different compression and holding combinations: IM without the holding pressure, IM with the holding pressure, ICM without the holding pressure, and ICM with the holding pressure: (**a**) COP material; (**b**) PMMA material.

The effect of compression on the average part mass was lower than 30 mg, which was negligible with respect to the effect of the holding pressure. In the case, the standard deviation was considered as a quantitative way to characterize the process precision; for both the materials, precision increased as holding was performed. An interaction between compression and holding was observed. The lowest precision was observed when performing ICM for PMMA (Figure 14b). Meanwhile, the most favorable condition for COP was observed for IM (Figure 14a).

When the process was optimized, the weight variation of ICM parts was reduced to 1 mg when a long compression gap and a low holding pressure were selected. This meant an increment in the precision of three times the previous IM result.

**Figure 15:**
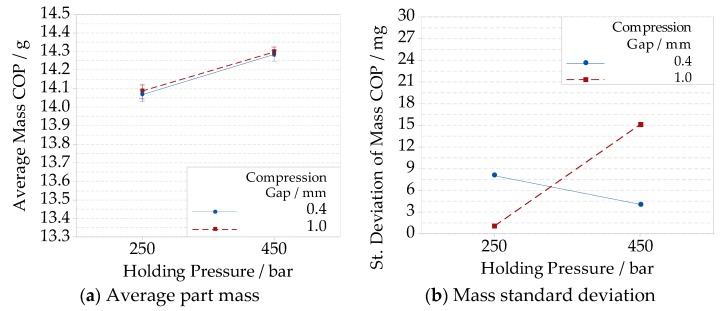
Interaction plots showing for the COP material, and different compression gap and holding pressure levels combinations: (**a**) average part mass; (**b**) mass standard deviation.

Increasing the holding pressure with a short compression gap resulted in a mass increment from (14.067 ± 0.008) g to (14.282 ± 0.004) g (i.e., +1.5% increase), and from (14.088 ± 0.001) g to (14.298 ± 0.015) g (i.e., +1.5% increase) in the case of long compression gap, see (Figure 15a). The interaction between the compression gap and the holding pressure (Figure 15b) was not negligible when considering the mass standard deviation. An increment of the holding pressure caused a reduction of the mass standard deviation when the compression gap was kept short. The opposite phenomenon was observed with the long compression gap.

During compression, the action on the polymer inside the cavity works as an in-thickness force. On the other hand, during holding, the polymer is pushed from the injection nozzle through the injection point. The two actions on the polymer are of different natures, and the final filling conditions depend on the combined action of the two phases. The significance of the interaction of holding and compression was verified on both at the micro-level and at the global part level with their corresponding quality features.

## 4. Conclusions

The evaluation of the manufacturing signature of IM and ICM for different quality features in Fresnel lenses manufacturing was performed, investigating the replication of absolute dimensions, part warpage, injection pressure, and part mass in order to identify (both locally, i.e., at the optical micro feature level, and globally, i.e., at the part level). The most suitable quality criteria and manufacturing fingerprints for the optimization of the production have been determined and validated. The methodology is based on a quantitative metrological approach, and it allows for the comparison of the performance of IM and ICM on the experimental case of micro-structured optics. As a result, the adequate selection and setting of process parameters was found, enabling specified quality features to be achieved, in terms of both accuracy and precision. The main conclusions of the research can be summarized as follows:

- The replication of absolute dimensions in terms of groove step height and pitch improved from IM to ICM. A higher replication fidelity was achieved using the PMMA material. The compression phase had a larger influence on pitch values. An optimal condition for ICM was achieved when a higher holding pressure and a lower compression gap were selected.
- The holding phase was of paramount importance in both IM and ICM for the reduction of the warpage. The parts’ warpage was described with second-order polynomials and it was related to differential shrinkages of the parts, due to different process conditions. The absence of the holding phase in ICM was detrimental. It increased the warpage and favored the formation of air traps, as shown in the case when processing PMMA. The optimal condition of ICM promoting less warpage occurred when a high holding pressure level was selected.
- The compression phase led to a pressure cavity variation over time, both during and after the filling phase. However, the main driver of pressure variation during the filling phase was the compression gap while its effect during the holding phase was overcome by the holding pressure. *P_work_* was used as an indicator of the energy transferred to the polymer part during processing, and its monitoring served as a production manufacturing signature.
- The holding phase was the major contributor to variations in the average part mass. In this case, IM and ICM showed similar process precision. However, process precision, measured as a global part mass standard deviation, can be minimized in the optimized ICM case with a high compression gap and low holding phase levels.

From these conclusions, it was shown that ICM leads to advancements in terms of surface micro-replication, part warpage and process precision with respect to IM. However, particular attention has to be paid in setting the holding phase and the compression gap when carrying out the ICM process, in order to achieve high replication fidelity, as well as the required geometrical accuracy and precision.

## Figures and Tables

**Table 1:** Measurement set-up of the laser scanning confocal microscope for the studied Fresnel lens.

Objective		Image Properties	
Field of view	640 × 640 µm^2^	Dimensions	3712 × 640 µm^2^
Numerical aperture	0.6	Image size	5328 × 913 pixels^2^
XY diffraction limit	0.412 µm	X pixel size	0.120 µm
Stitching overlap	20%	Y pixel size	0.701 µm
Z spatial resolution	0.030 µm		

**Table 2:** Step height measurement uncertainty budget for COP and PMMA in injection molding (IM) and ICM, of a nominal grooves’ height of 17.3 µm. Measurements are calibrated with a gauge step of 14.45 µm.

Uncertainty Contributor	IM—COP	ICM—COP	IM—PMMA	ICM—PMMA
*u_cal,z_* (calibration artefact)	0.26 µm	0.26 µm	0.26 µm	0.26 µm
*u_p,z_* (instrument repeatability)	0.03 µm	0.03 µm	0.03 µm	0.03 µm
*u_b,z_* (instrument thermal)	0.00 µm	0.00 µm	0.00 µm	0.00 µm
*u_wp,z_* (part repeatability)	0.13 µm	0.13 µm	0.11 µm	0.11 µm
*u_wt,z_* (part thermal)	0.01 µm	0.01 µm	0.04 µm	0.04 µm
*u_form,z_* (form error)	0.17 µm	0.17 µm	0.03 µm	0.05 µm
*k* (coverage factor)	2	2	2	2
*U* (exp. Uncertainty)	0.7 µm	0.7 µm	0.6 µm	0.6 µm

**Table 3:** Pitch measurements’ uncertainty budget for COP and PMMA in IM and ICM, of a nominal grooves’ width of 748.1 µm. Measurements are calibrated with a gauge block thickness of 1500 µm.

Uncertainty Contributor	IM—COP	ICM—COP	IM—PMMA	ICM—PMMA
*u_cal,z_* (calibration artefact)	0.08 µm	0.08 µm	0.08 µm	0.08 µm
*u_p,z_* (instrument repeatability)	1.09 µm	1.09 µm	1.09 µm	1.09 µm
*u_b,z_* (instrument thermal)	0.01 µm	0.01 µm	0.01 µm	0.01 µm
*u_wp,z_* (part repeatability)	1.12 µm	1.12 µm	1.43 µm	1.43 µm
*u_wt,z_* (part thermal)	0.45 µm	0.45 µm	0.83 µm	0.84 µm
*u_form,z_* (form error)	0.99 µm	1.45 µm	0.73 µm	1.27 µm
*k* (coverage factor)	2	2	2	2
*U* (exp. Uncertainty)	3.8 µm	4.4 µm	4.2 µm	4.7 µm

**Table 4:** Screening experiments investigating the effect of compression and holding phases as independent process stages of IM and ICM for PMMA and COP materials: (**a**) with three factors on two levels; (**b**) constant parameters.

(a)			(b)	
Factors	Low Level	High Level	Parameters	Value
**Compression**	OFF	ON	**Injection Velocity**	40 mm/s
**Holding**	OFF	ON	**Switch/over**	10 mm
**Material**	PMMA	COP	**Compression gap**	0.7 mm
			**Holding pressure**	450 bar
			**T melt COP**	280 °C
			**T melt PMMA**	260 °C
			**T mold COP**	105 ± 3 °C
			**T mold PMMA**	93 ± 3 °C

**Table 5:** Optimization experiment investigating the effect of compression gap and holding pressure in the ICM of the COP material: (**a**) with two factors on two levels; (**b**) constant parameters.

(a)			(b)	
Factors	Low Level	High Level	Parameters	Value
**Compression gap**	0.4 mm	1.0 mm	**Injection velocity**	40 mm/s
**Holding pressure**	250 bar	450 bar	**Switchover**	10 mm
			**T melt COP**	280 °C
			**T mold COP**	105 ± 3 °C

**Table 6:** Results summary of part warpage as the maximum absolute deviation in the evaluated area, and as a quadratic coefficient of regression of the evaluated residuals.

Factors	Max Warpage µm	Quadratic Regression Coefficient µm/mm^2^	Factors	Max Warpage µm	Quadratic Regression Coefficient µm/mm^2^
IM without holding; COP	2.0	−0.6	ICM; 250 bar, 0.4 mm	1.1	0.3
IM; COP	0.7	0.2	ICM; 250 bar, 1.0 mm	2.0	0.4
ICM without holding; COP	4.3	0.9	ICM; 450 bar, 0.4 mm	1.0	0.2
ICM; COP	2.0	0.4	ICM; 450 bar, 1.0 mm	1.1	0.2
IM without holding; PMMA	1.6	0.4			
IM; PMMA	1.9	0.5			
ICM without holding; PMMA	106.9	39.5			
ICM; PMMA	2.2	0.6
